# Uncoupling cell division and cytokinesis during germline development in metazoans

**DOI:** 10.3389/fcell.2022.1001689

**Published:** 2022-11-03

**Authors:** Abigail R. Gerhold, Jean-Claude Labbé, Ramya Singh

**Affiliations:** ^1^ Department of Biology, McGill University, Montréal, QC, Canada; ^2^ Institute for Research in Immunology and Cancer (IRIC), Montréal, QC, Canada; ^3^ Department of Pathology and Cell Biology, Université de Montréal, Succ. Centre-ville, Montréal, QC, Canada

**Keywords:** incomplete cytokinesis, intercellular bridges, germ cells, germline development, metazoan

## Abstract

The canonical eukaryotic cell cycle ends with cytokinesis, which physically divides the mother cell in two and allows the cycle to resume in the newly individualized daughter cells. However, during germline development in nearly all metazoans, dividing germ cells undergo incomplete cytokinesis and germ cells stay connected by intercellular bridges which allow the exchange of cytoplasm and organelles between cells. The near ubiquity of incomplete cytokinesis in animal germ lines suggests that this is an ancient feature that is fundamental for the development and function of this tissue. While cytokinesis has been studied for several decades, the mechanisms that enable regulated incomplete cytokinesis in germ cells are only beginning to emerge. Here we review the current knowledge on the regulation of germ cell intercellular bridge formation, focusing on findings made using mouse, *Drosophila melanogaster* and *Caenorhabditis elegans* as experimental systems.

## Main text

### Introduction

Cytokinesis is the last step of cell division during which the two daughter cells become physically separated. It starts during anaphase, with the formation of a contractile actomyosin ring that ingresses between the two nascent daughter cells. Closure of this ring gives rise to a transient intercellular bridge, which is severed during abscission. Most cell divisions end in complete cytokinesis and abscission, and disruptions to the cytokinetic machinery are associated with several diseases, including cancer (reviewed in Lacroix and Maddox, 2012; Lens and Medema, 2019). Some cells, however, undergo regulated incomplete cytokinesis and remain connected after cell division by a stable intercellular bridge. There is a growing appreciation for the prevalence of stable intercellular bridges in multicellular eukaryotes, suggesting that they serve recurrent roles and may even have contributed to the evolution of multicellularity (Chaigne and Brunet, 2022).

Stable intercellular bridges are ubiquitous in animal germ lines. Early ultrastructural study of mammalian testes (cat; Burgos and Fawcett, 1955) and fetal ovaries (rabbit; Zamboni and Gondos, 1968) revealed that both male and female developing germ cells are connected by intercellular bridges. Evidence of germ cell intercellular bridges was subsequently found in diverse species across the animal phylogeny (reviewed in Chaigne and Brunet, 2022), including in humans (Fawcett et al., 1959; Ruby et al., 1970a), mice (Ruby et al., 1969; Huckins and Oakberg, 1978), frogs (Ruby et al., 1970b; Kloc et al., 2004), fish (Bertho et al., 2021), chick (Skalko et al., 1972; Ukeshima and Fujimoto, 1991), fruit flies (Fawcett et al., 1959; Brown and King, 1964), round worms (*Caenorhabditis elegans*, Hirsh et al., 1976; *Ascaris lumbricoides*, Foor, 1967), segmented worms (*Diopatra cuprea*, Anderson and Huebner, 1968; leeches and earthworms, Świątek et al., 2009) and Cnidaria (*Hydra*, Alexandrova et al., 2005; Fawcett et al., 1959). Germ cell intercellular bridges range in size from 0.5 to 10 µm in diameter (reviewed in Haglund et al., 2011) and are generally large enough to permit the free passage of macromolecules and organelles (e.g., Zamboni and Gondos, 1968). Due to the presence of these relatively large connections, most animal germ cells develop within syncytial cysts.

Although many of the molecular regulators required for cytokinesis are conserved in animals, the precise composition of germ cell intercellular bridges varies between species and even between the sexes of the same species. In addition, while most germ lines are syncytial, different syncytial architectures are observed. Thus, despite being a deeply conserved feature of animal germ lines, the mechanisms by which germ cell intercellular bridges form may be diverse. Here, we first describe the possible roles of germ cell intercellular bridges and the variety of syncytial organizations observed. We then highlight the common themes and key differences in the formation of germ cell intercellular bridges, using three well-studied model systems–the *Mus musculus* (mouse) testis, the *Drosophila melanogaster* (*Drosophila*) ovary and the *Caenorhabditis elegans* (*C. elegans*) hermaphrodite gonad.

### Germ cell intercellular bridges likely serve several purposes

The widespread occurrence of germ cell intercellular bridges implies that they play an important role in gamete production. Indeed, disruption of germ cell intercellular bridges generally leads to reduced fecundity or sterility (Yue and Spradling, 1992; Xue and Cooley, 1993; Greenbaum et al., 2006; Greenbaum et al., 2009; Ikami et al., 2021). Early ultrastructural studies that provided evidence for both cytoplasmic and organelle sharing within germ cell cysts, led to three main hypotheses regarding the role of germ cell intercellular bridges. Bridges could 1) support synchronous germ cell development (Fawcett et al., 1959); 2) ensure phenotypic equivalence between genetically distinct haploid gametes (Erickson, 1973); or 3) allow some cells within the cyst to act as ‘nurse’ cells (Brown and King, 1964; Ruby et al., 1969). These hypotheses are not mutually exclusive, and all three are supported by more recent work, suggesting that germ cell intercellular bridges serve several functions, the necessity of which may depend on the particular mode of gametogenesis.

Germ cell development often includes synchronous mitotic divisions, meiotic entry and/or maturation and the sharing of signals *via* intercellular bridges may permit this. For example, loss of germ cell bridges in the mouse testis disrupts the synchronous development of spermatogonial cells (Rezende-Melo et al., 2020), and in fetal mouse ovaries, cytoplasmic sharing *via* intercellular bridges is required for the coordinated transition to meiosis within cysts (Soygur et al., 2021). However, not all syncytial germ lines exhibit strict synchronous development. For example, in the syncytial *C. elegans* adult gonad, mitotic divisions are only loosely clustered within the pool of mitotic germ cells, and adjacent mitotic cells divide asynchronously (Maciejowski et al., 2006; Gerhold et al., 2015; Zellag et al., 2021). Similarly, the border between the mitotic and meiotic regions of the adult *C. elegans* gonad is not sharp and mitotic and meiotic cells are interspersed (Hansen et al., 2004; Crittenden et al., 2006).

Germ cell development can also include phases of haploid gene expression and sharing of these gene products *via* intercellular bridges may be important for maintaining phenotypically equivalent gametes, and thus Mendelian patterns of inheritance. For example, the study of haploid gene products during spermatogenesis in mammals revealed that they transit *via* intercellular bridges and are shared between germ cells within the cyst (Braun et al., 1989; Ventela et al., 2003). Haploid-expressed gene products that evade sharing can confer a selective advantage for the sperm that carry them and distort gene inheritance patterns (Veron et al., 2009). Recently, single-cell RNA sequencing of mammalian haploid spermatids showed that, although the majority of allelic differences are erased by sharing of haploid gene products *via* intercellular bridges, some incompletely shared products can act as selfish genetic elements (Bhutani et al., 2021). Therefore, maintaining phenotypically equivalent gametes is likely a major evolutionary pressure in favor of germ cell intercellular bridges, at least in species with haploid gene expression.

Finally, in females, there is extensive evidence that intercellular bridges support “nursing” of developing oocytes by other germ cells within the cyst. In meroistic ovaries, a subset of germ cells acts as nurse cells by donating cytoplasm and organelles to the future oocyte before undergoing programmed cell death. This mode of oogenesis is well-documented in many animals, including *Drosophila* and other insects (reviewed in Mahajan-Miklos and Cooley, 1994; Telfer, 1975), mouse (Pepling and Spradling, 2001; Lei and Spradling, 2016; Niu and Spradling, 2022), clitellate annelids (reviewed in Świątek and Urbisz, 2019), and *C. elegans* (Gumienny et al., 1999; Wolke et al., 2007). However, some panoistic ovaries, in which all germ cells become oocytes and nurse cells are not found, are also syncytial (e.g. stoneflies; reviewed in Buning, 1993), suggesting an alternate role for intercellular bridges in these female germ lines.

The function of intercellular bridges may also be influenced by the regulation of bridge traffic. For example, in the *Drosophila* ovary, transport through intercellular bridges is selective and unidirectional, from nurse cells to the developing oocyte (Bohrmann and Biber, 1994; Lu et al., 2021). Selective transport and/or restricted diffusion through intercellular bridges has also been observed in germ cell cysts in the *Drosophila* and mouse testis (Ventela et al., 2003; Veron et al., 2009; Kaufman et al., 2020). Thus, germ cell intercellular bridges are not necessarily passive conduits for cytoplasmic and organelle sharing; rather the regulation of transport through intercellular bridges is likely to play a key role in determining their function.

### Germline syncytia come in a variety of forms

**FIGURE 1:**
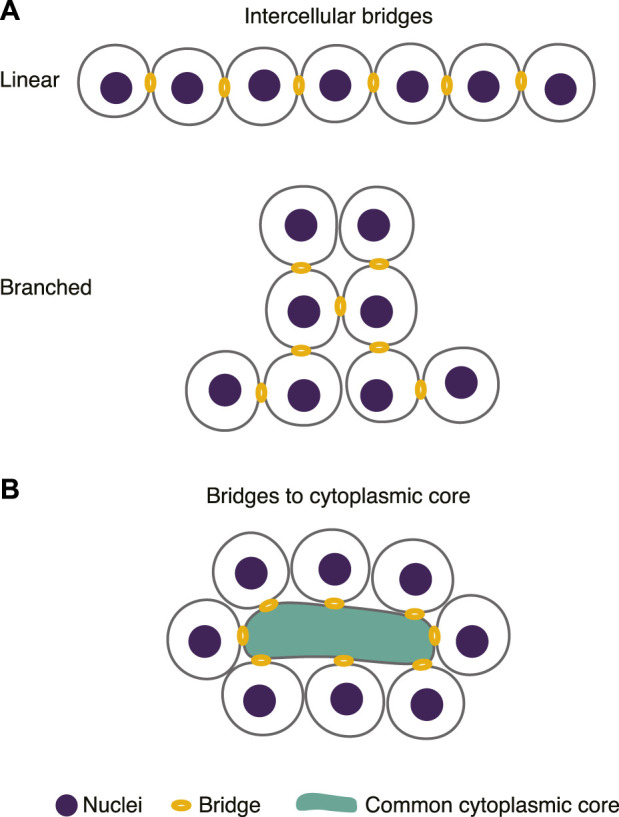
Germline syncytia vary in architecture. **(A)** Germline syncytia formed by intercellular bridges that directly connect germ cells. These syncytia can be linear (upper) or branched (lower), depending on the number of bridges per cell. **(B)** Germline syncytia formed by cytoplasmic bridges that connect germ cells to a common cytoplasmic core.

While the full diversity of animal germline syncytia awaits classification, within those that have been described, two main syncytial architectures can be found: 1) germ cell cysts in which cells are connected directly by an intercellular bridge; and 2) germ cell cysts in which each germ cell possesses a single intercellular bridge connecting it to an anucleate cytoplasmic core (Figure 1). For simplicity we will refer to both as “intercellular” bridges. This difference has important implications for how germ cell intercellular bridges are formed during cell division. In the first class, incomplete cytokinesis, followed by stabilization and maturation of the residual intercellular bridge between dividing germ cells, would support cyst formation, with each cell division producing one new bridge. In the second, cell division needs to produce two bridges such that each daughter cell retains a connection to the common cytoplasmic core of the cyst.

Germ cell cysts that fall into the first class can be roughly divided into two types: cysts with a linear arrangement of germ cells and cysts with a branched arrangement (Figure 1A). In linear cysts, each germ cell contains no more than two intercellular bridges and the cyst forms as an unbranched chain. This arrangement is common in mammalian testes (Dym and Fawcett, 1971; Huckins, 1971; Oakberg, 1971; reviewed in Yoshida, 2010), and has also been found in the ovaries of some polychaetous annelids (Anderson and Huebner, 1968). In branched cysts, the number of intercellular bridges per a germ cell is variable. For example, cysts in the *Drosophila* ovary are highly branched. Cysts are composed of sixteen germ cells with an invariant pattern of fifteen intercellular bridges; eight cells have one intercellular bridge, four have two, two have three and two have four (Brown and King, 1964). Various branched configurations are also found in the ovaries of *Xenopus* (Kloc et al., 2004) and mice (Lei and Spradling, 2016).

Germ cell cysts that fall into the second class (Figure 1B) are found in nematodes (e.g., *Pristionchus pacificus*, Rudel et al., 2005; *Ascaris lumbricoides*, Foor, 1967; *C. elegans*, Hall et al., 1999, Hirsh et al., 1976) and are common within clitellate annelids and flat worms (reviewed in Świątek and Urbisz, 2019). Here the number of cells per a cyst can vary widely. For example, in *C. elegans* hermaphrodites, each of the two gonad arms comprises a single cyst of approximately 1000 germ cells (Kimble and White, 1981), which supports continuous gamete production throughout the reproductive period. In comparison, the ovaries of the white earthworm, *Enchytraeus albidus*, contain several smaller cysts, each with its own cytoplasmic core (Urbisz et al., 2017). Variations on this architecture are also found in mites where oocytes are arranged around and connected to a large central cell called the ovarian nutritive cell (reviewed in Witalinski, 2014), and in the ovaries of many true bugs (Hemiptera), where nurse cells and developing oocytes are spatially and developmentally segregated but remain connected by a central core of cytoplasm (Kugler et al., 2006; reviewed in Buning, 1993).

### Germ cell intercellular bridges form during cell division by modifications to cytokinesis

Stable germ cell intercellular bridges typically arise from incomplete cytokinesis. Here we will briefly outline the cytokinetic steps most relevant to intercellular bridge formation.

In animal cells, cytokinesis fundamentally rests on actin and non-muscle myosin forming a contractile ring at the cell cortex, that ingresses between the separated sets of sister chromatids at the end of anaphase (reviewed in D'Avino et al., 2015; Green et al., 2012). Accurate positioning and assembly of the contractile ring depends on signals emanating from the mitotic spindle. Astral microtubules promote the relaxation of cortical tension at the spindle poles, while the central spindle, an array of antiparallel microtubules that forms between separating chromosomes, and spindle microtubules from opposite spindle poles, stimulate contractile ring formation at the equatorial cortex. Contractile ring formation requires the activation of the small GTPase RhoA by the centralspindlin complex, a heterotetramer of a Rho GTPase-activating protein (GAP), MgcRacGAP, and a kinesin-like protein, mitotic kinesin-like protein 1 (MKLP1; see Table 1). RhoA locally triggers the activation of downstream effectors such as formins (actin nucleators), Rho kinase (myosin activator) and the scaffold protein Anillin to promote the assembly and ingression of the contractile actomyosin ring.

**TABLE 1:** Regulators of cytokinesis and/or germ cell intercellular bridge formation^a^.

Mouse	*Drosophila*	*C. elegans*	Brief description
RhoA	Rho1	RHO-1	Small GTPase, master regulator of cytokinesis
Ect2	Pebble	ECT-2	RhoA GTPase Exchange Factor (GEF)
MgcRacGAP	Tumbleweed	CYK-4	GTPase Activating Protein (GAP), centralspindlin complex component
MKLP1	Pavarotti	ZEN-4	Kinesin, centralspindlin complex component
mDia1, 2, 3	Diaphanous	CYK-1	Formin, actin nucleator
Rock1, 2	DRok	LET-502	Rho Kinase, myosin activator
Anln	Anillin	ANI-1, ANI-2	Anillin, actomyosin scaffold protein
SEPT2, 7, 9	Peanut, Sep1, Sep2	UNC-59, UNC-61	Septins
TEX14	-	-	CEP55 interactor, germ cell intercellular bridge formation in mice
CEP55	-	-	Midbody and abscission scaffolding protein in mice
*Usp8*	USP8	*USP-50*	Deubiquitinase, ring canal formation in *Drosophila*
*CD2AP, CIN85*	Cindr	*CDAP-2*	Actin-binding adaptor, ring canal stability in *Drosophila*
*Add1, 2, 3*	Hts-RC	*ADD-1, ADD-2*	Adducin, actin-binding protein, ring canal maturation in *Drosophila*
*Klhl12*	Kelch	*KEL-1*	Cullin 3 complex substrate adaptor, ring canal maturation in *Drosophila*

^a^ The regulators for which the functional homolog has not been unambiguously ascribed and/or implicated in germ cell intercellular bridge regulation have been italicized. Relevant references can be found in the main text.

Constriction of the contractile ring culminates in the formation of a transient intercellular bridge centered on the midbody. The midbody comprises the microtubule-dense central spindle remnant encircled by a stable cortical ring called the midbody ring, which is enriched in proteins including Anillin, myosin, MgcRacGAP and MKLP1 (reviewed in Carim et al., 2020; Mierzwa and Gerlich, 2014; Peterman and Prekeris, 2019). The midbody coordinates the progressive disassembly of cytoskeletal components, including microtubule depolymerization and the removal of actomyosin filaments, and the recruitment of the endosomal sorting complexes required for transport (ESCRT) machinery. Two flanking rings form on either side of the midbody, which include Anillin, Septins and actomyosin, to further constrict the intercellular bridge (Hu et al., 2012; Wang et al., 2019). Assembly of ESCRT III filaments at one of the two secondary constrictions narrows the bridge and eventually leads to membrane scission, separating sister cells and releasing the midbody remnant (reviewed in Stoten and Carlton, 2018).

The formation of stable intercellular bridges in animal germ lines relies on modifications to cytokinesis that block its completion. The next sections focus on the mechanisms by which incomplete cytokinesis leads to intercellular bridge formation in the germ lines of mouse, *Drosophila* and *C. elegans*.

### Intercellular bridges in the mouse testis

**FIGURE 2:**
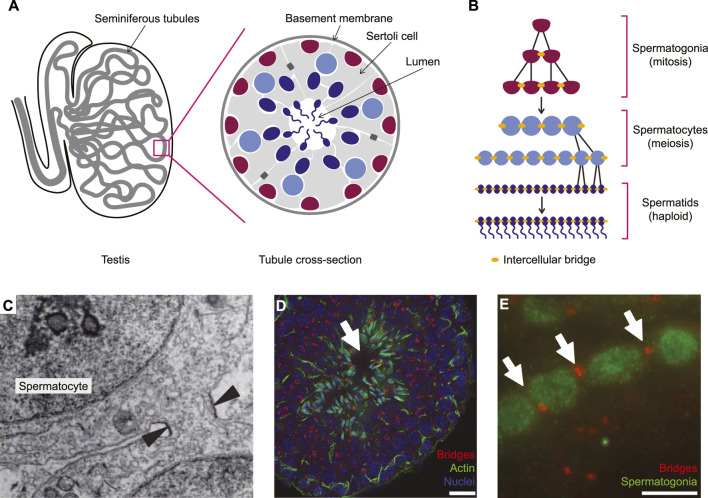
Intercellular bridges during spermatogenesis in the mouse testis. **(A)** Spermatogenesis occurs within the convoluted seminiferous tubules and is polarized, with mitotic spermatogonia at the basal surface and maturing spermatids at the luminal surface. **(B)** Both mitotic and meiotic divisions during spermatogenesis are incomplete and germ cells form long synchronous chains until individuation as mature sperm. **(C)** An electron micrograph showing a stable intercellular bridge (arrowheads) between two spermatocytes in the rat testis. Reproduced with permission from Dym and Fawcett, 1971. **(D)** A cross-section of a mouse seminiferous tubule stained for TEX14 to mark stable intercellular bridges (red), actin (green) and nuclei (DAPI, blue). Intercellular bridges are evident throughout the tubule. Arrow indicates the lumen. Scale bar is 25 μm. **(E)** A higher magnification view showing TEX14-labelled intercellular bridges (red; arrows) between spermatogonia (green) in the mouse testis. Scale bar is 10 μm. **(D)** and **(E)** were adapted with permission from Greenbaum et al., 2006, Copyright (2006) National Academy of Sciences, United States.

As in all mammals, sperm development in the mouse takes place in the seminiferous tubules, which form long, convoluted loops within the testis (Figure 2A; reviewed in Yoshida, 2016). Each tubule contains germ cells embedded within an epithelium of specialized somatic cells, called Sertoli cells, that line the central lumen of the tube. Germ cell differentiation is polarized along the apical-basal axis of this epithelium, with mitotic germ cells, the spermatogonia, adjacent to the basement membrane and maturing haploid spermatozoa at the apical surface, adjacent to the lumen (Figure 2A). Germ cells are connected throughout development by stable intercellular bridges, which form by incomplete cytokinesis during both mitotic and meiotic divisions (Figure 2B). As a result, germ cells develop as long, synchronous chains of cells from the early spermatogonia stage until individuation and release into the lumen as spermatozoa (reviewed in Greenbaum et al., 2011).

Although stable intercellular bridges are present throughout spermatogenesis (Figures 2C,D), their size and composition change during germ cell development (Greenbaum et al., 2007). In early spermatogonia, bridge diameter is less than 1 µm (Greenbaum et al., 2007). Bridge diameter increases slightly as spermatogonia mature and differentiate to spermatocytes, before increasing approximately 2-fold to a final diameter of 2–3 µm in spermatids (Greenbaum et al., 2007). Bridges joining spermatogonia and spermatocytes contain an outer ring composed of the centralspindilin complex, MgcRacGAP and MKLP1, and three septins, SEPT2, SEPT7 and SEPT9, but not Anillin, surrounding an inner ring containing Testis-expressed gene 14 (TEX14), Centrosomal 55-kDa protein (CEP55), and pericentrin (Greenbaum et al., 2007; Chang et al., 2010; Iwamori et al., 2010). As bridges mature, the inner ring grows to merge with the outer ring and septins are removed (Greenbaum et al., 2007). Other proteins that are associated with intercellular bridges during mouse spermatogenesis include delta-tubulin (Kato et al., 2004), RNA binding motif protein 44 (RBM44; Iwamori et al., 2011), heat-shock factor 2 (HSF2; Greenbaum et al., 2006), ectoplasmic specialization-associated protein KIAA121, topoisomerase 2-beta (TOP2B), and the tight junction protein Zonula occludens-1 (ZO1; Iwamori et al., 2020). However, the roles these proteins play in bridge formation or stabilization are not well understood. Finally, several intercellular bridge components in rat testes are also considered conserved in mice, including F-actin (Russell et al., 1987), protocadherin α3 (Johnson et al., 2004) and plectin (Guttman et al., 1999).

TEX14 was the first essential component of the stable intercellular bridge in mice testes to be identified (Greenbaum et al., 2006). *Tex14* mutant male mice are sterile, with seminiferous tubules containing markedly few late meiotic and post-meiotic germ cells that lack intercellular bridges (Greenbaum et al., 2006). TEX14 also regulates intercellular bridges in the mouse ovary (Ikami et al., 2021; Niu and Spradling, 2022) and mutations in *Tex14* are associated with infertility in pigs and humans (Gershoni et al., 2017; Sironen et al., 2017; Fakhro et al., 2018), consistent with a fundamental role for this protein in the regulation of stable intercellular bridges, at least in mammals.

In the mouse testis, TEX14 promotes intercellular bridge formation by blocking abscission (Iwamori et al., 2010). Dividing germ cells in the mouse testis form midbodies containing microtubules, Anillin, the centralspindlin complex and Septins, similarly to cells that undergo complete cytokinesis (Greenbaum et al., 2007; Greenbaum et al., 2009). The only known difference is the presence of TEX14 during late cytokinetic furrow ingression (Figure 2E). TEX14 bears a short motif (AxGPPx3YxPP) that is also found in tumor susceptibility gene 101 (TSG101), a component of the ESCRT I complex, and ALG-2 interacting protein X (ALIX), an ESCRT adaptor protein (Morita et al., 2007; Lee et al., 2008; Iwamori et al., 2010). In cultured somatic cells, this motif enables TSG101 and ALIX to interact with CEP55 at the midbody in late cytokinesis, which in turn promotes the recruitment of ESCRT III complex components to mediate abscission (Carlton and Martin-Serrano, 2007; Morita et al., 2007; Carlton et al., 2008; Lee et al., 2008). The same motif in TEX14 was shown to promote its interaction with CEP55, allowing it to compete with TSG101 and ALIX for CEP55 binding, precluding the loading of ESCRT III regulators at the midbody, and thus effectively blocking abscission (Iwamori et al., 2010; Kim et al., 2015). Indeed, expressing portions of TEX14 in cultured somatic cells is sufficient to compromise the loading of ALIX at the midbody and the completion of abscission in some, but not all, cells (Iwamori et al., 2010; Kim et al., 2015).

Several mechanisms contribute to the function of TEX14 at intercellular bridges. Recruitment of TEX14 to the midbody occurs during telophase and is thought to rely on MKLP1 (Greenbaum et al., 2007). This local increase in TEX14 levels relatively early during midbody formation favors its interaction with CEP55 and blocks the recruitment of ALIX and TSG101 (Iwamori et al., 2010; Kim et al., 2015). In addition, the affinity of TEX14 for CEP55 is higher than that of ALIX or TSG101, and its dissociation rate from CEP55 is ∼15 times slower (Iwamori et al., 2010; Kim et al., 2015). This combination of increased affinity for and slower dissociation from CEP55, together with high levels of TEX14 at the midbody and its early recruitment, allows TEX14 to prevent CEP55 from interacting with ALIX and TSG101 and to thereby block the completion of abscission.

Thus, in the mouse testes, germ cell cytokinesis proceeds through midbody formation, but fails at abscission, due to the presence of TEX14. The midbody is then converted into a stable intercellular bridge (Greenbaum et al., 2007). However, while TEX14 expression is sufficient to block abscission in some somatic cells (Iwamori et al., 2010; Kim et al., 2015), it does not cause these somatic cells to develop stable germ cell-like intercellular bridges (Greenbaum et al., 2007), and the mechanism by which the midbody is transformed into a stable intercellular bridge remains to be uncovered. In addition, although CEP55 is required for abscission in cultured cells (Fabbro et al., 2005; Zhao et al., 2006; Morita et al., 2007), recent studies using knockout *Cep55* mice demonstrated that abscission occurs in the absence of CEP55 in many, if not most, cell types (Tedeschi et al., 2020; Little et al., 2021; Zhang et al., 2021). Whether these alternative routes to abscission function in germ cells and, if so, whether their inhibition also relies on TEX14, has not been addressed. Finally, TEX14 and CEP55 are mainly found in vertebrates (Chaigne and Brunet, 2022) and thus the regulation of intercellular bridges in the germ lines of most animals likely relies on other regulators.

### Ring canals in the *Drosophila* female ovary

**FIGURE 3:**
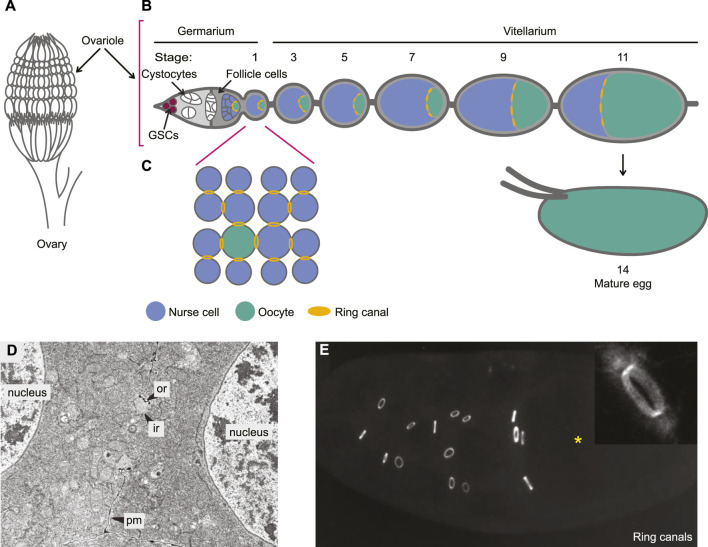
Ring canals during oogenesis in the *Drosophila* ovary. **(A)** Each ovary in female *Drosophila* contains 16–23 ovarioles. **(B)** Oogenesis is polarized along the length of each ovariole, with germline stem cells (GSCs) and mitotic cystocytes in the anterior germarium, maturing egg chambers arrayed along the vitellarium, and mature eggs (stage 14) at the posterior. **(C)** Cystocyte divisions are incomplete, giving rise to a 16-cell cyst that is maximally branched, with cystocytes connected by stable intercellular bridges called ring canals. The oocyte develops from one of the two cystocytes with the most ring canals. The remaining cells differentiate as nurse cells. **(D)** An electron micrograph showing a ring canal between two nurse cells. The ring canal forms a break in the plasma membrane (pm) and is composed of an inner (ir) and outer (or) rim. **(E)** A stage 10 egg chamber with ring canals immunostained for hu-li tai shao (Hts). The oocyte is indicated with an asterisk. Insert shows the actin staining pattern for a ring canal from an egg chamber of the same stage. **(D)** and **(E)** adapted with permission from Robinson et al., 1994, The Company of Biologists, Ltd.

Germ cell intercellular bridges have been studied extensively in the *Drosophila* ovary, in part owing to the outstanding genetic tools available in this model organism and the large size of these bridges in later stages of development. *Drosophila* females have two ovaries, each housing 16–23 tube-like ovarioles, depending on the strain (Figure 3A; Sarikaya et al., 2012). Egg production occurs in an assembly-line fashion along the length of each ovariole, with germline stem cells at the anterior tip and mature eggs at the posterior (Figure 3B; reviewed in Bastock and St Johnston, 2008). The anterior region of the ovariole is called the germarium. Within the germarium, germline stem cells divide asymmetrically to produce a cystoblast, which undergoes four rounds of synchronous mitotic divisions with incomplete cytokinesis, generating a cyst of sixteen germ cells, referred to as cystocytes. The cystocytes are interconnected by stable intercellular bridges called ring canals. Due to a stereotypic pattern of division orientation, these cysts are maximally branched (reviewed in de Cuevas et al., 1997). One of the two cystocytes that bear four ring canals will develop into the oocyte, while the remaining cells become nurse cells (Figure 3C). As the cyst develops, it is enveloped by a single layer of supporting somatic cells called follicle cells, eventually forming a spheroidal Stage 1 egg chamber that exits the germarium. Egg chambers mature as they move through the posterior part of the ovariole, called the vitellarium, until they reach Stage 14, when oocyte growth and maturation are complete. In each germarium there are several germ cell cysts at different stages of development and in each vitellarium there are seven or eight maturating egg chambers.

Ring canals form by incomplete cytokinesis during the mitotic phase of cyst growth, while ring canal maturation and growth occur largely after mitotic cystocyte divisions are complete (Ong and Tan, 2010; reviewed in Haglund et al., 2011). Through the first three rounds of cystocyte mitosis, ring canals are enriched in late cytokinetic regulators, such as actin, Anillin, the centralspindilin subunit Pavarotti (Pav/MKLP1) and the actin-binding adaptor Cindr/CD2AP, and were thus thought to be arrested cytokinetic rings (Field and Alberts, 1995; de Cuevas and Spradling, 1998; Minestrini et al., 2002; Haglund et al., 2010). The appearance of phosphotyrosine (pY) epitopes at ring canals in 8-cell cysts is one of the first molecular features that differentiates ring canals from cytokinetic rings (Robinson et al., 1994). After the fourth and final round of mitosis, ring canal maturation starts in earnest with recruitment of the *hu-li tai shao* gene product Hts-RC, dissociation of Cindr/CD2AP, and formation of an actin-rich inner rim (Warn et al., 1985; Theurkauf et al., 1993; Robinson et al., 1994; Tilney et al., 1996; Petrella et al., 2007; Haglund et al., 2010). Further changes include the gradual disappearance of Anillin (Field and Alberts, 1995), the arrival of Kelch, an actin binding protein and substrate adaptor for the Cullin-3 E3 ubiquitin ligase complex (Xue and Cooley, 1993; Robinson et al., 1994; Kelso et al., 2002; Hudson and Cooley, 2010) and the accumulation of E-cadherin complexes that help anchor and stabilize ring canals at the membrane during tissue growth (Loyer et al., 2015). Ring canal maturation is accompanied by a massive increase in ring size, from a <1 µm diameter in the 16-cell cyst to ∼10 µm in diameter in stage 11 egg chambers (Figures 3D,E; Ong and Tan, 2010; Robinson et al., 1994; Tilney et al., 1996; Warn et al., 1985).

While ring canal maturation has been well characterized, how cytokinesis is initially arrested to allow ring canals to form is less well understood. Neither CEP55 nor TEX14 are found in *Drosophila*; thus a different molecular mechanism must enable incomplete cytokinesis in this system. Recent evidence suggests that the gene *Usp8* is key to ensuring incomplete cytokinesis within *Drosophila* germline cysts (Mathieu et al., 2022). USP8 is a deubiquitinase, a class of enzymes that typically catalyze the removal of ubiquitin moieties from substrate proteins. Downregulation of *Usp8* in *Drosophila* egg chambers leads to cystocytes that undergo complete abscission, resulting in the formation of germline cysts with fewer than 16 cells. Overexpressing USP8 in cells that normally undergo complete cytokinesis blocks abscission and causes ectopic cyst formation. USP8 deubiquitinates the ESCRT III components CHMP2B and CHMP4 and prevents their accumulation at the intercellular bridge. This change in the timely recruitment of ESCRT III regulators is likely key to impairing abscission and enabling subsequent ring canal formation.

Thus, rather than being derived from a stalled cytokinetic ring, as was formerly proposed (Robinson and Cooley, 1997; Haglund et al., 2011), germ cell intercellular bridges in the *Drosophila* ovary are more likely derived from the midbody following stalled abscission. This idea is supported by recent live-imaging work showing full constriction of the cytokinetic ring into a midbody-like structure, which then resolves into a ring canal with an open lumen, in both male and female cystocytes (Price et al., 2022). Therefore, the formation of germ cell intercellular bridges in *Drosophila* and mice is more similar than previously thought, with both involving mechanisms that regulate ESCRT III and thereby block abscission. In the absence of abscission, the midbody is then remodeled into a stable intercellular bridge.

### Rachis bridges in the *C. elegans* hermaphrodite germ line

**FIGURE 4:**
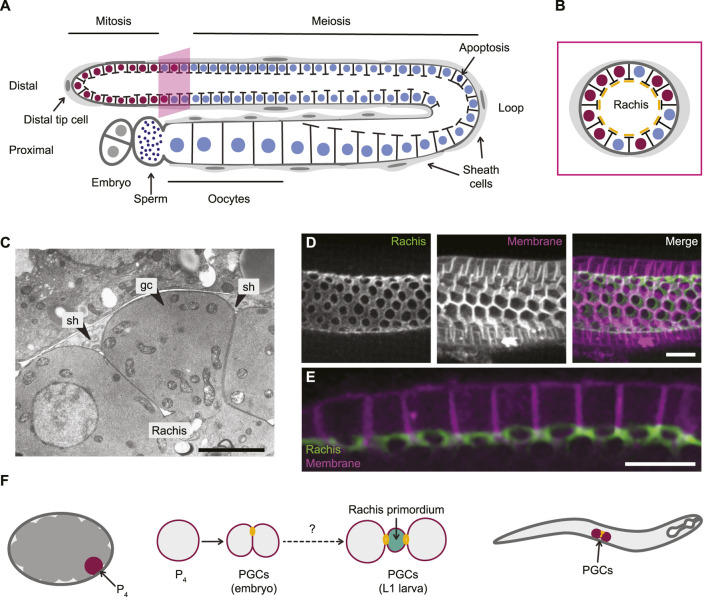
Rachis bridges in the *C. elegans* hermaphrodite gonad. **(A)** The *C. elegans* hermaphrodite gonad is composed of two U-shaped tubes. Mitotic germ cells at the distal tip of each arm enter meiosis as they move proximally, and maturing oocytes are found at the proximal tip, adjacent to the spermatheca. **(B)** Germ cells are arranged in a rough monolayer around a common core of cytoplasm called the rachis. Each germ cell maintains a stable rachis bridge connecting it to the rachis. **(C)** An electron micrograph of a cross section through the gonad showing a germ cells (gc) with an open rachis bridge. Somatic sheath cells (sh) contact germ cells basally. Scale bar is 5 μm. Adapted with permission from Hall et al., 1999, Elsevier. **(D)** A maximum intensity projection of ∼half of the adult gonad tube, showing the rachis surface (green; marked by ANI-2:GFP) and cell membranes (magenta; marked by mCherry:PH^PLCδ^). **(E)** A longitudinal section of the adult gonad showing the rachis bridges (green; marked by ANI-2:GFP) connecting each germ cell to the rachis. Cell membranes are in magenta. **(D)** and **(E)** are adapted from Priti et al., 2018, CC-BY. Scale bars are 10 μm. **(F)** All germ cells are derived from the P_4_ germline precursor cell that gives rise to the two primordial germ cells (PGCs) during embryogenesis. P_4_ undergoes incomplete cytokinesis and the two embryonic PGCs remain connected by a stable intercellular bridge. By an unknown mechanism, the connection between the two PGCs is transformed into the rachis primordium during late embryogenesis, such that, by the time the L1 larva hatches, each PGC possesses its own rachis bridge.

In adult *C. elegans* hermaphrodites, the germ line is found within two U-shaped gonad arms, with gametogenesis occurring in an assembly line-like fashion along the distal-proximal axis of each arm (Figure 4A; reviewed in Hubbard and Schedl, 2019). Each gonad arm is capped at its distal end by a so-called Distal Tip Cell that establishes a niche for the underlying pool of mitotically dividing germline stem and progenitor cells. Germ cells that exit the niche enter meiotic differentiation and progress through gametogenesis, which is completed in the proximal region of each gonad arm. Many germ cells are also fated to undergo apoptosis, which is restricted to a region of the gonad just before the bend in the ‘U’ (Gumienny et al., 1999). *C. elegans* hermaphrodites are more accurately described as self-fertile females. Their first ∼300 gametes differentiate as sperm, which are used for self-fertilization and constitute their only male characteristic. Gametogenesis then irreversibly switches to oocyte production.

The distal gonad arm is a blind-ended tube, with germ cells arranged into a rough monolayer around a central core of cytoplasm, termed the rachis (Figures 4A,B). Each germ cell maintains a single cytoplasmic bridge that connects it to the rachis (Figure 4C; Hall et al., 1999; Hirsh et al., 1976). The rachis serves as a conduit for cytoplasmic streaming, which carries material (e.g. protein, mRNA and organelles) from germ cells in the distal arm to maturing oocytes at the proximal end of the tissue (Wolke et al., 2007). Thus, in *C. elegans*, all germ cells likely serve, at least transiently, as nurse cells. Although all germ cells have an intercellular bridge (Amini et al., 2014), the size of bridges varies as germ cells progress through gametogenesis. Bridges within the mitotic zone average ∼2 µm in diameter (Rehain-Bell et al., 2017). Bridges expand to 3–4 µm in diameter as germ cells enter meiosis, shrink in the bend region, where many cells undergo apoptosis, expand again to 4–5 µm in diameter as oocytes mature, before closing completely when oocytes cellularize (Rehain-Bell et al., 2017; Lee et al., 2018). How bridge dynamics are coordinated with gametogenesis is not known but may be relevant for the regulation of germ cell apoptosis (Raiders et al., 2018; Chartier et al., 2021).

In *C. elegans*, germ cell intercellular bridges are known as rachis bridges or ring channels. Each bridge is kept open by a stable actomyosin ring that is similar in composition to the cytokinetic ring (Figures 4D,E). Both F-actin and non-muscle myosin II (NMY-2) localize to rachis bridges, as do two isoforms of Anillin (ANI-1 and ANI-2), the septin UNC-59, the centralspindlin components ZEN-4 (MKPL1) and CYK-4 (MgcRacGAP), the Rho GEF ECT-2, LET-502 (ROCK) and the formin CYK-1 (Maddox et al., 2005; Zhou et al., 2013; Amini et al., 2014; Lee et al., 2018; Priti et al., 2018). Most of these factors are also found lining the rachis surface between rachis bridges, forming a tissue-level contractile network that is under tension (Figure 4D; Priti et al., 2018), and depletion of any of them results in varying degrees of germ line disorganization and sterility (Piekny and Mains, 2002; Maddox et al., 2005; Zhou et al., 2013; Amini et al., 2014; Lee et al., 2018; Priti et al., 2018).

While rachis bridges are similar in organization and composition to cytokinetic rings, there are some notable differences. Myosin turnover is slower in rachis bridges, suggesting that rachis bridges are more stable structures (Priti et al., 2018). CYK-4/MgcRacGAP is also stably associated with rachis bridges and the rachis surface, and, unlike during cytokinesis, neither its localization nor its function seem to require ZEN-4/MKLP1 (Zhou et al., 2013; Lee et al., 2018). Instead, it has been proposed that CYK-4/MgcRacGAP is enriched at the rachis independently of ZEN-4/MKLP1 and microtubules, where it promotes RhoA activation during oocyte cellularization (Lee et al., 2018). ZEN-4/MKLP1, which is also found at the rachis surface, may contribute to germ line microtubule organization (Zhou et al., 2013).

For rachis bridges to remain stably open, one or more regulators of contractility must be present to prevent closure of the actomyosin ring. Several factors have been proposed to fill this role. ANI-2 is an atypical anillin, which lacks the predicted myosin and actin binding domains, and which may serve to counterbalance the pro-contractile activity of the canonical anillin, ANI-1, specifically in the germ line (Maddox et al., 2005; Amini et al., 2014). Correspondingly, rachis bridges are smaller in *ani-2* mutants, and this phenotype is partially rescued by depletion of ANI-1 (Amini et al., 2014). Two additional interactors of ANI-1 were recently identified that may also counteract bridge closure. The Ste20-family germinal center kinase GCK-1, and its binding partner cerebral cavernous malformation 3 (CCM-3), localize to the rachis and are enriched on rachis bridges, where they are thought to antagonize ANI-1 and/or restrict the recruitment of NMY-2 (Pal et al., 2017; Rehain-Bell et al., 2017). Direct regulation of myosin activity may also be important, as depletion of the myosin phosphatase regulatory subunit MEL-11, which should increase myosin activity, results in smaller rachis bridges (Priti et al., 2018), while depletion of the myosin activating kinase LET-502 (ROCK) results in larger bridges (Rehain-Bell et al., 2017; Priti et al., 2018).

While several candidates have been identified as important for maintaining syncytial architecture in the mature germ line, how this structure originates and how it expands during germ line development is poorly understood. All *C. elegans* germ cells arise from a single germ cell precursor, called P_4_, that is born during embryogenesis following a series of asymmetric divisions (Figure 4F). P_4_ divides symmetrically to give rise to the two primordial germ cells (PGCs), termed Z_2_ and Z_3_ (Hirsh et al., 1976; Deppe et al., 1978; Sulston et al., 1983). P_4_ cytokinesis is incomplete and leaves a stable intercellular bridge that directly connects the two PGCs, although this bridge is initially either too small or obstructed to allow for cytoplasmic exchange (Amini et al., 2014; Goupil et al., 2017). Thus far, no clear molecular mechanism has emerged that would explain why cytokinesis is incomplete in P_4._ Similar to germ cells in *Drosophila* and mice, furrow ingression is normal in P_4_ and a midbody-like structure appears to form, but abscission does not occur (Goupil et al., 2017). How abscission is inhibited is not known. As is the case for *Drosophila*, *C. elegans* does not possess homologs of TEX14 or CEP55, and thus the mechanism of incomplete abscission must rely on other regulators. *C. elegans* is predicted to possess an ortholog of USP8 (USP-50) but its role in germ line function has not been investigated.

The two PGCs remain mitotically quiescent for the remainder of embryogenesis and only resume cell cycle progression after first instar (L1) larvae have hatched and begun feeding (Kimble and White, 1981). At hatching however, the PGCs are no longer connected to one another by a single intercellular bridge, but rather each has its own cytoplasmic bridge connecting it to the nascent rachis (Figure 4F; Bauer et al., 2021). It is unclear whether the stable intercellular bridge present after the division of P_4_ is inherited by one of the two PGCs, or if the two bridges have been synthesized *de novo*. PGCs undergo significant cortical remodeling during embryogenesis, including formation of polar lobes that are stabilized by actomyosin rings (Abdu et al., 2016; Maniscalco et al., 2020) which could conceivably provide a source for additional rachis bridge material. Extensive live imaging of primordial germ line development during embryogenesis will be needed to properly assess the events that nucleate syncytial organization.

The number of germ cells greatly increases during larval development, from the two PGCs found in L1 larvae to the ∼2000 germ cells contained within the two gonad arms in adult animals (Hirsh et al., 1976; Kimble and White, 1981). The syncytial architecture of the *C. elegans* germ line poses a distinct challenge for cell division–a mother cell with a single connection to a common cytoplasm must produce two daughter cells, each with its own individual connection to this same shared cytoplasmic core. How dividing germ cells achieve this remains unclear, although recent advances indicate several possible mechanisms.

A first model was proposed by Świątek, et al., in 2009, after electron microscopy micrographs of dividing germ cells in several clitellate annelids showed the ingressing cytokinetic ring contacting and seemingly bisecting the stable ring that connected the mother cell to the cytophore (the equivalent structure to the rachis in nematodes). Based on these results, Świątek concluded that daughter cells could each inherit a stable connection to the cytophore if 1) the cytokinetic ring is anchored to the existing stable cytophore bridge and ingresses asymmetrically towards it; and 2) the cytokinetic ring bifurcates and partitions the existing cytophore bridge between daughter cells. Conceptually this is similar to what has been observed during PGC formation in *Drosophila* embryos, where two contractile rings exist simultaneously in cellularizing PGCs, one dividing the initial PGC bud from the embryo syncytium and the other cleaving the bud into two PGCs after mitosis (Cinalli and Lehmann, 2013).

A second view emerged from analysis of fixed germ lines from late larval and adult hermaphrodites, which showed that the diameter of rachis bridges is reduced in mitotic germ cells to a size that precludes resolution of an open lumen (<0.3 µm, Seidel et al., 2018). The cytokinetic furrow appears to ingress towards the rachis surface, and in nascent daughter cells, small bridges become visible in adjacent pairs, an arrangement that was also observed following germ cell division in clitellate annelids (Świątek et al., 2009). These observations could suggest that bridge duplication in *C. elegans*, like that seen in clitellate annelids, occurs *via* bisection by the cytokinetic ring, albeit at a highly reduced bridge diameter (Seidel et al., 2018). More recently, however, live imaging of PGC divisions in L1 larvae showed that the cytokinetic ring ingresses at an angle relative to the stable rachis bridge, which, unlike in adults, remains sufficiently open to permit cytoplasmic diffusion (Bauer et al., 2021). The physical gap between the closing cytokinetic ring and the rachis surface decreases progressively, and the cytokinetic ring and/or its constituents eventually integrate into the rachis. Whether the differences between these two observations are due to developmental factors and/or tissue characteristics or are the result of different imaging approaches awaits resolution.

**FIGURE 5:**
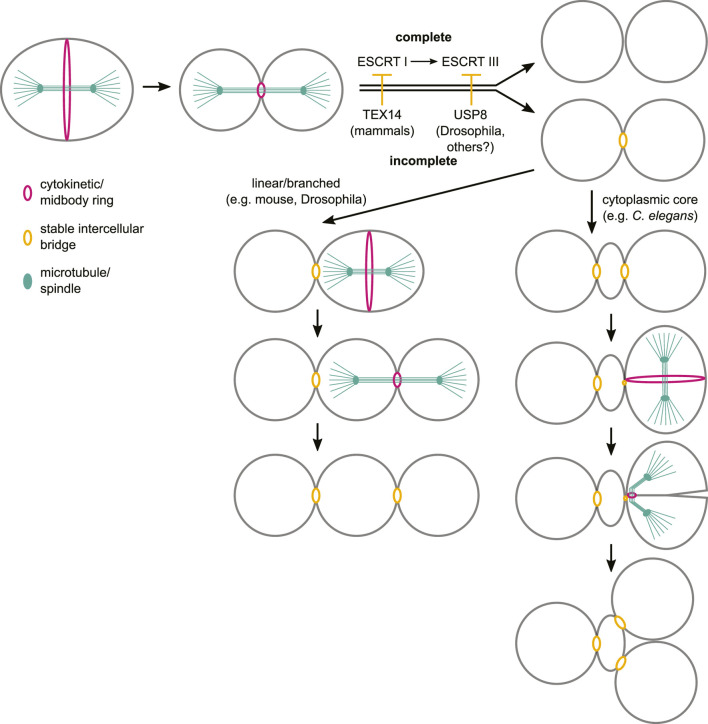
Stable intercellular bridge formation in germ cells by incomplete cytokinesis. In metazoans, stable intercellular bridges (orange) arise from the cytokinetic/midbody ring (magenta) following incomplete germ cell cytokinesis, through mechanisms that impede the activity of ESCRT components during abscission. These events are reiterated in germ cells that develop within linear and branched cysts (e.g., mouse, *Drosophila*), thus enabling cyst expansion. In architectures where germ cells are connected to an anucleate cytoplasmic core (e.g., *C. elegans*), the stable intercellular bridges must undergo duplication through a mechanism that is currently unknown. We propose that subsequent germ cell divisions within this architecture also occur by incomplete cytokinesis, in a manner that allows one daughter cell to inherit the pre-existing stable intercellular bridge while the other daughter inherits the bridge arising of the cytokinetic/midbody ring. This mechanism requires further experimental validation and could also rely on bisection of the stable intercellular bridge by the cytokinetic ring, as was proposed previously (Świątek et al., 2009).

Together, these results suggest a model for bridge duplication during germ cell division in *C. elegans*. First, the incomplete cytokinesis program that is initiated in the germline precursor P_4_ remains active and thus cytokinetic ring ingression is not followed by abscission, similarly to intercellular bridge formation in the mouse testis and the *Drosophila* ovary (Figure 5). The persistence of CYK-4/MgcRacGAP and ZEN-4/MKLP1, known components of the midbody, further supports the idea that rachis bridges may be derived from midbody rings. Second, cytokinetic ring closure occurs towards the rachis surface and is followed by membrane rearrangements that enable the stabilized midbody ring to connect one of the daughter cells to the rachis, while the other daughter cell inherits the original rachis bridge. Exit from mitosis then promotes the relaxation of actomyosin contractility and, thereby, the reopening of the two rachis bridges. Alternatively, the cytokinetic ring could divide the existing rachis bridge in two, as proposed for bridge duplication in clitellate annelids. However, the highly reduced rachis bridge diameter in adult mitotic germ cells, and the physical separation between the cytokinetic ring and existing rachis bridge in mitotic PGCs, suggest that bridge duplication in clitellate annelids and *C. elegans* may not be equivalent, despite similar syncytial architectures. Whether this model holds true awaits additional experimentation to determine the precise mechanism of abscission inhibition and the manner of rachis bridge duplication.

### Conclusions and future perspectives

The nearly universal presence of stable intercellular bridges in animal germ lines suggests that they are fundamentally important for fertility. However, similarly to other aspects of germ line development (e.g., the timing and mode of germ line specification; Whittle and Extavour, 2017), cross-species comparisons have uncovered substantial differences in bridge function and form. Germ cell intercellular bridges play a variety of roles, are diverse in molecular composition and generate syncytia of various architectures, raising the question of what, if anything, they have in common. Despite this diversity, bridge formation is tied to the deeply conserved processes of cell division and cytokinesis and could be correspondingly constrained. In the three examples reviewed here, bridges form *via* incomplete cytokinesis, with cytokinetic arrest occurring late in the process, after midbody formation, at or near the time when ESCRT regulators are typically loaded to coordinate abscission. A common theme in germ cell bridge formation may therefore be inhibition of abscission, with molecular mechanisms specifically adapted to the regulation of normal abscission in each species (Figure 5). Obstructed abscission has also been proposed to underlie intercellular bridge formation more generally (e.g., in embryonic stem cells; Chaigne et al., 2020), and, as ESCRT regulators are highly conserved in eukaryotes (Chaigne and Brunet, 2022), it will be interesting to determine whether this is a broadly used mechanism to control complete versus incomplete cytokinesis.

Blocking abscission alone does not appear to be sufficient for intercellular bridge formation. As noted above, overexpressing TEX14 prevents abscission in some somatic cells, but these cells do not develop stable intercellular bridges (Greenbaum et al., 2007; Iwamori et al., 2010; Kim et al., 2015). Similar outcomes have been observed in *Drosophila* S2 cells; when abscission was inhibited by interfering with ESCRT III activity, cells stayed connected, but did not form bridges (El Amine et al., 2013). Thus, a second possible commonality in germ cell intercellular bridge formation is the conversion of the midbody into a stable intercellular bridge (Price et al., 2022). The mechanism (or mechanisms) by which the midbody is transformed into a stable intercellular bridge is not known and is an important area for future research. Work in this area may also uncover additional layers of regulation that impact late cytokinesis in cells that divide completely and/or that can be co-opted to drive cytokinesis failure, and thus contribute to chromosome instabilities in diseased states, such as cancer.

Understanding how intercellular bridge dynamics are coordinated with cell cycle regulation and germ cell development is also a topic that merits further investigation. In *C. elegans*, and other species in which germ cells are arranged around a common cytoplasmic core, cell division brings the stable intercellular bridge in close contact with the dynamic cytokinetic ring. How cells reconcile these two contractile structures is unclear. Even in germline cysts with a branched or linear arrangement of cells, cell division occurs in the presence of at least one stable intercellular bridge, which must maintain its distinct contractile properties as the rest of the cell cortex is remodeled in preparation for cytokinesis. It will be interesting to determine how germ cell intercellular bridges are affected by and maintained through cell division. Finally, germ cell development culminates in the production of individual gametes; yet how intercellular bridges close to accommodate gamete maturation and individuation is poorly understood.
